# Standard Splenic Volume Estimation in North Indian Adult Population: Using 3D Reconstruction of Abdominal CT Scan Images

**DOI:** 10.1155/2011/707325

**Published:** 2011-03-08

**Authors:** Adil Asghar, Dushyant Agrawal, S. M. Yunus, P. K. Sharma, S. H. H. Zaidi, Aruna Sinha

**Affiliations:** ^1^Hamdard Institute of Medical Sciences and Research, New Delhi 110062, India; ^2^Department of Anatomy, JNMC, AMU, Aligarh, India; ^3^TMMC and RC, Moradabad 244001, India; ^4^KGMU, Lucknow 226003, India; ^5^RMCH, Bareilly 243006, India

## Abstract

A prospective study was carried out to establish normative data for splenic dimensions in North Indian population and their correlation with physical standard on abdominal CT of 21 patients aged between 20 and 70 years having no splenic disorders. Splenic volume was measured by two methods—volume and surface rendering technique of Able 3D doctor software and prolate ellipsoid formula. Volumes measured by both the techniques were correlated with their physical standards. Mean splenic volume was 161.57 ± 90.2 cm^3^ and range 45.7–271.46 cm^3^. The volume of spleen had linear correlation with body height (*r* = 0.512, *P* < .05). Splenic volume (cm^3^) = 7 × height (cm) − 961 can be used to generate normal standard volume of spleen as a function of body height in North Indian population (with 95% confidence interval). This formula can be used to objectively measure the size of the spleen in adults who have clinically suspected splenomegaly.

## 1. Introduction

Evaluation of splenic size by palpation can be extremely inaccurate because spleen is never palpable till it is enlarged 2 to 3 times its own size [1]. Determination of spleen size is important in diagnosing small, normal or enlarged spleens. Splenomegaly is an important clinical sign for diagnosing varieties of diseases, for example, portal hypertension, glycogen storage disorder, hematological malignancy, and other disorders [2, 3]. In the past, various clinical and radiological techniques (USG and nuclear medicine) have been used to estimate organ volumes. Technique for determining splenic volume by ultrasonography had been presented in various studies [4]. Unfortunately, volume determination by 2D USG can be inaccurate because of the variable, irregular contour of spleen and overlapping of splenic outline by bone, bowel gas, or left kidney [5]. New 3D reconstruction of CT images is more accurate than 2D ultrasonography [6–8].

 Our purpose was primarily to document the normal range of various dimension of spleen (volume and surface area) in North Indian adults and to study the relationship of these splenic dimensions with different physical parameters of patients. Till now, we did not have any normative data of normal splenic volume in North Indian population based on CT-measurements. So, we have tried to generate normative data which could be used for research tool and in certain clinical settings in which objective determination of splenomegaly is required.

## 2. Materials and Methods

CT scans of 21 patients (12 male and 9 female) were used to measure the volume of spleen. The age of patients ranged from 20 to 70 years (50.33 ± 18.9 years, Table 1). The data was collected prospectively from December 2006 to April 2007 with permission from Department of Radiodiagnosis, KGMU, Lucknow, and informed consent from each patient was taken. CT scans were done for various clinical presentations, followup cases of abdominal trauma or pain. The patient's body weight and height were recorded at the time of the CT examination. Axial and cross-sectional images of spleen were collected from a computer attached to helical CT scan machine. The technical parameters were 120 kv potential, 120 mA current, and 10 mm slice width with identical reconstruction index and rotation time of 1.5 secs. The medical records of all patients were reviewed. Patients whose spleen appeared abnormal on CT scans were excluded. Additionally, any patient who had clinical, biochemical, or radiological evidences of any condition that could affect the size of the spleen, for example, hematological disorders, abdominal malignancies, infection and portal hypertension, splenic trauma, cyst, and autoimmune diseases were excluded from the study.

**Figure 1:**
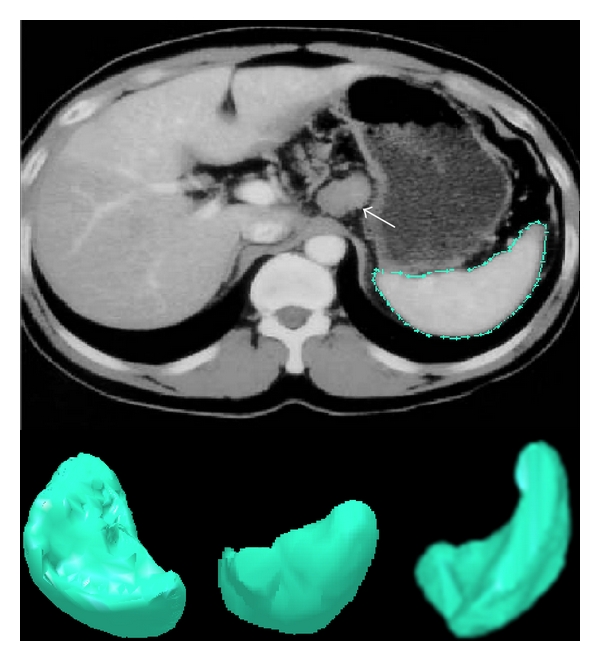
3D reconstructed image of spleen.

The volume and surface area of spleen were measured by volume and surface rendering technique of Able 3D-doctor software by analysis of CT Images. Volume and surface rendering techniques of this software are a computerized program to create 3D image of any organ from stacks of cross-sectional images of that organ in a CT/MRI film. Stacks of images in CT film of each patient were opened in Able 3D-doctor software. Spleen was identified in each cross-section and longitudinal section of CT-scan images. Spleen boundary was outlined digitally in each section. Sections having maximum length, width, and thickness were also recorded as length, width, and thickness of spleen in that CT film. This software had created 3D picture of spleen (Figure 1). Then, with the help of software, volume and surface area were recorded (called as observed volume). Also, we calculated the volume of spleen manually by using the standard clinical prolate ellipsoid equation for spleen [0.524 × splenic index (max. length × max. width × max. thickness)] [8–10].

### 2.1. Statistical Analysis

All statistics were generated by SPSS version 10. Student's *t*-test was used for comparison of mean between the two sexes. *P* < .05 was considered significant for comparison of means and for regression analysis. Association between splenic parameters (volume, surface area) and physical standards of patients were assessed with the Pearson correlation coefficient. To identify the exact pattern of relationship, nonlinear regression as well as linear regression was applied. Multiple regression analysis were applied in backward stepwise fashion to test the independent effect of all physical standards on splenic parameters.

## 3. Results

**Figure 2:**
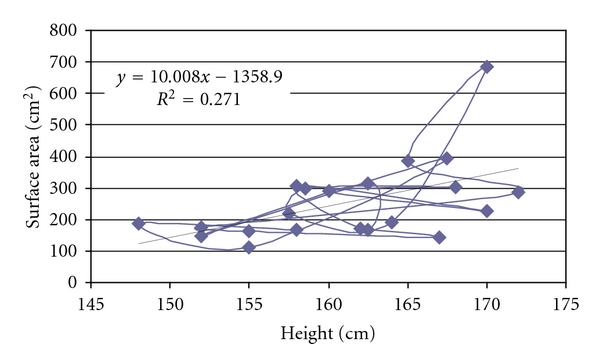
Scatter-plot to demonstrate the correlation between height of patient and surface area of spleen.

**Figure 3:**
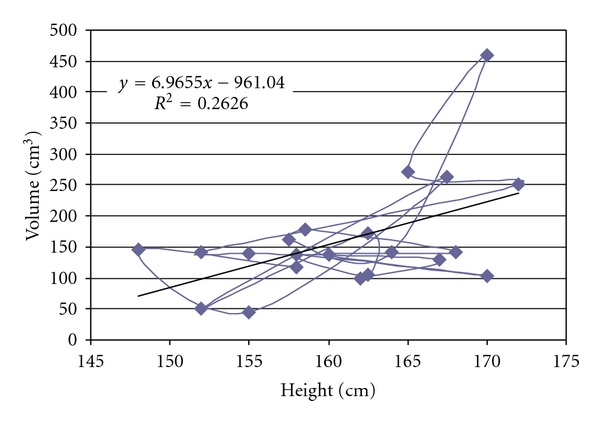
Scatter-plot to demonstrate the correlation between volume of spleen and height of patient.

**Figure 4:**
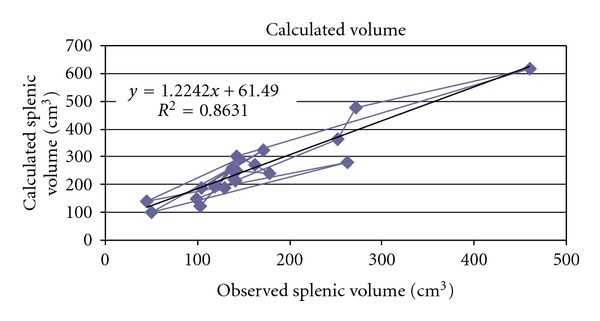
Scatter-plot to demonstrate the correlation between calculated volume by prolate ellipsoid formula and observed volume by 3D reconstruction of spleen.

The mean splenic dimensions were 161.57 ± 90.2 cm³ in volume and 254.01 ± 127.56 cm² in surface area by volume and surface rendering techniques (Table 2). There was significant correlation between height and surface area (*r* = 0.521, *P* < .05, Figure 2) and splenic volume (*r* = 0.512, *P* < .05, Figure 3). Volume calculated by prolate ellipsoid formula was 259.22 ± 118.92 cm³, and this also significantly correlated with true volume measured by volume and surface rendering techniques (*r* = 0.929, *P* < .001, Figure 4).

## 4. Discussion

A wide variety of imaging modalities, including conventional radiography, nuclear scan, ultrasonography, CT scan and MR scan have been used to study the spleen. Computed tomography has been considered as a reliable modality to study the spleen or other intra-abdominal organs in vivo. In studies comparing CT volumetric measurements by summation-area technique of spleen in cadavers or patients prior to splenectomy, with the corresponding actual volume determined by water displacement, 3–5% mean error was observed. Helical CT and volume rendering technique used in this study abolished error due to respiratory excursions and manual tracing and provided more accurate data [12]. Various other previous studies were analyzed and their data extrapolated with the current study to see any difference (Table 3) [7, 11–20]. Differences among these studies were due to different methods used by authors and on different populations or races. Spielmann et al. (2005) found that volume of spleen as well as all linear splenic dimensions were well correlated with participants, height (*r* = 0.4, *P* < .0002) [20]. Many literatures were available that were showing linear correlation between patients height and linear splenic dimensions [4, 18, 21–23]. In our study, we observed splenic volume 161.57 ± 90.2 cm³ (female 118.39 ± 47.7 cm³ and male 192.29 ± 99.3 cm³, *P* > .05) using 3D reconstruction. This observed splenic volume was best correlated with height (*r* = 0.512, *P* < .05) and we had found linear regression which formulated as *volume (cm³) = 6.965 *×* height (cm) *−* 961.04*. Hoefs et al. (1999) calculated splenic volume in healthy volunteers was 201 ± 77 cm³ through liver-spleen scan by CT and MRI. They did not find any significant difference in the two sexes (male 189 ± 82 cm³ and female 214 ± 68 cm³). They found linear correlation of splenic volume with age and suggested formula of splenic volume = 335 − 4.05 × age (*r* = 0.548, *P* < .05) [16]. But in our study we found that splenic volume also moderately correlated with age (*r* = 0.4, *P* < .05) and body surface area (BSA) (*r* = 0.433, *P* < .05).

The calculated splenic volume by prolate ellipsoid formula was 259.29 ± 118.86 cm³ (female 217.44 ± 70.92 cm³ and male 288.36 ± 141.26 cm³ *P* > .05) and it showed a weak correlation with height (*r* = 0.39, *P* < .05). This calculated volume was strongly correlated with observed volume measured by 3D CT reconstruction (*r* = 0.929, *P* < .0001) and formulated the correction through the linear regression as *calculated volume (cm³) = 1.224 × observed volume (cm³) + 61.49*. Prassopoulos et al. (1997) studied on 140 patients of different age groups with normal spleen and found that the product of *L* × *W* × Th (splenic index) best correlated with true volume measured by summation-area technique on CT scan (*r* = 0.94, *P* < .001). They formulated best on linear regression *S* Vol. = 30 + 0.58 (*W* × *L* × Th) in cm³ [12]. Difference between them and our study might be due to consideration of cephalocaudal length along 10th rib in axial CT image by us whereas they had measured longitudinal length. 

 We had calculated surface area of spleen: 254.01 ± 127.56 cm² (female: 205.56 ± 77.65 cm² and male: 290.35 ± 14.78 cm², *P* > .05). We did not have any data for comparison. Surface area of spleen was best correlated with body height (*r* = 0.521, *P* < .05) and BSA (*r* = 0.452, *P* < .05). It had linear regression with both height and BSA (Figure 2).

## 5. Conclusion

**Figure 5:**
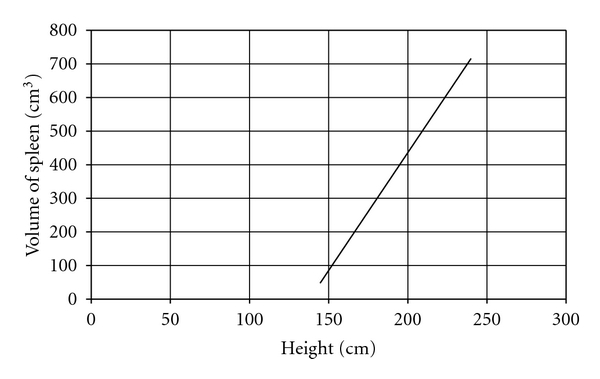
Regression nomogram: volume of spleen versus height.

Establishing normal parameters is mandatory for defining the pathological changes in size of spleen in routine sonography or CT investigation. Our data supported the normal range for spleen dimension given by different authors on different populations. These data can be used to avoid the false positive diagnosis of splenomegaly. These normative data of normal splenic volume in adults can be used as a research tool in certain clinical situation in which objective measurement of splenic dimensions and comparisons with standard of normal splenic volume would be useful. For calculation of splenic volume, we found that values observed by prolate ellipsoid formula and 3D reconstruction technique were significantly different. So, we can correct this following formula to get true volume of spleen which developed through linear regression model: *0.524 × splenic index (length × width × thickness) = 1.224 × observed volume + 61.49 or observed or true volume (cm³) = 0.43 × splenic index *−* 50.23.* Almost all dimension of spleen had best correlation with a patient's height. So, the normative data of volume of spleen could be generated with these formulae. *Volume of spleen (cm³) = 6.965 *×* height (cm) *−* 961* (Figure 5) and *Surface area of spleen (cm²) = 10 *×* height (cm) *−* 1358.9.* In our study, we conclude that dimensions of spleen in North Indian population best correlates with height like European and American population, but, The coefficient of correlation is moderately stronger than other populations or races.

## Figures and Tables

**Table 1:** Physical standard of patients.

Sex	Physical standard of patients					
	Age (yrs.)	Weight (kg.)	Height (cm)	Body surface area (m²)	Body mass index	Numbers of patients
Male	51.33 ± 18.82	65.4 ± 9.9	165.45 ± 4.4	1.72 ± 0.13	24 ± 3.22	12
Female	49 ± 12.18	55.22 ± 4.38	155.4 ± 4.3	1.56 ± 0.035	23.12 ± 1.43	9

Total	No significant difference (*P* > .05)					21

**Table 2:** Mean value and standard deviation of dimensions of spleen.

Methods of measurement	Male (Mean ± SD)	Female (Mean ± SD)	Total (Mean ± SD)
(1) Volume rendering technique (cm³)	192.29 ± 99.3	118.39 ± 47.7	161.57 ± 90.2
(2) Prolate ellipsoid formula (cm³)	288.36 ± 141.26	217.44 ± 70.92	259.29 ± 118.86
(3) Surface area by surface rendering technique (cm²)	290.35 ± 14.78	205.56 ± 77.65	254.01 ± 127.56

Significance level	*P* > .05 between two sexes		

**Table 3:** Comparisons of volume of spleen in different population and by different techniques.

Authors	Techniques	Population	Mean volume of spleen (cm³)	Range (cm³)
Liu et al. [11]	Multidetector CT scan	Chinese	190.94 ± 70.37	—
Prassopoulos et al. [12]	Summation-area technique (CT scan)	European	214.6	107.2–314
Mazonakis et al. [13]	Random marking on MR scan	European	208.0 mL	115–293.6 mL
	Manual planimetry on MR scan	European	204.8 mL	117.9–289.8 mL
Hidaka et al. [14]	3D USG	Japanese	104 mL	—
Zhang et al. [15]	Radionuclide scan	—	185	—
Hoefs et al. [16]	Liver-spleen scan	American	201 ± 77	—
Henderson et al. [17]	Summation-area of CT scan	European	209 ± 76	—
Picardi et al. [18]	USG	European	140 mL	60–200 mL
Loftus et al. [19]	Water displacement	European	110 ± 70	26–250
Lamb et al. [7]	USG & CT –prolate ellipsoid	European	—	107–314
Spielmann et al. [20]	USG-prolate ellipsoid	American	333.6 ± 116.1	—
